# Brain activity underlying auditory perceptual learning during short period training: simultaneous fMRI and EEG recording

**DOI:** 10.1186/1471-2202-14-8

**Published:** 2013-01-14

**Authors:** Ana Cláudia Silva de Souza, Hani Camille Yehia, Masa-aki Sato, Daniel Callan

**Affiliations:** 1 Universidade Federal de São João del-Rei, Ouro Branco, Brazil; 2 Universidade Federal de Minas Gerais, Belo Horizonte, Brazil; 3 ATR Neural Information Analysis Laboratories, Kyoto, Japan

**Keywords:** Neural plasticity, Attention and performance, Perceptual learning, Auditory perception, Simultaneous fMRI and EEG, Time-frequency analysis

## Abstract

**Background:**

There is an accumulating body of evidence indicating that neuronal functional specificity to basic sensory stimulation is mutable and subject to experience. Although fMRI experiments have investigated changes in brain activity *after* relative to *before* perceptual learning, brain activity *during* perceptual learning has not been explored. This work investigated brain activity related to auditory frequency discrimination learning using a variational Bayesian approach for source localization, during simultaneous EEG and fMRI recording. We investigated whether the practice effects are determined solely by activity in stimulus-driven mechanisms or whether high-level attentional mechanisms, which are linked to the perceptual task, control the learning process.

**Results:**

The results of fMRI analyses revealed significant attention and learning related activity in left and right superior temporal gyrus STG as well as the left inferior frontal gyrus IFG. Current source localization of simultaneously recorded EEG data was estimated using a variational Bayesian method. Analysis of current localized to the left inferior frontal gyrus and the right superior temporal gyrus revealed gamma band activity correlated with behavioral performance.

**Conclusions:**

Rapid improvement in task performance is accompanied by plastic changes in the sensory cortex as well as superior areas gated by selective attention. Together the fMRI and EEG results suggest that gamma band activity in the right STG and left IFG plays an important role during perceptual learning.

## Background

The fact that cortical representations in adult animals can be modified by experience has led to extensive research regarding the neurophysiological mechanisms of cortical plasticity [1,2]. It is apparent that the knowledge of how plasticity can be induced would be of great value in developing treatment for individuals with brain damage or to optimize learning strategies in a normal brain. The capacity of reorganization, at least partly, accounts for certain forms of learning. Learning comes in many forms, some of which are explicit memories of objects, sounds, events and some of which are implicit and nondeclarative. One form of implicit memory, perceptual learning, involves improving one’s ability with practice, to discriminate differences in the attributes of simple stimuli.

One of the most interesting aspects of human sensory perception is that it is not restricted to an early critical life period but can be improved with practice even in adulthood [3]. Relatively little is known about how practice influences the performance of human adults on basic discrimination tasks but the understanding of the physiological substrates of learning will help the development of perceptual training schemes. Most of the perceptual learning studies are directed to the visual system. A number of studies have worked on primitive visual features such as hyperacuity and contrast discrimination [4,5], orientation [6-8], direction of motion [9,10] and texture discrimination [11].

Compared with the investigations in the visual system, the examination of perceptual learning in the auditory system is still in maturation. In traditional psychoacoustic experiments, training has been used mainly for the purpose of reaching asymptotic performance. More recently in the literature of learning in the auditory system, there has been an increase of the potential application of auditory training in the treatment of communication disorders [12-14], perceptual expertise [15-17], rehabilitation of abnormal perception [18,19] and improvement of cognitive skills [20-22].

One important aspect of perceptual learning involves its relation to the amount of training. According to Demany [23] few weeks of practice and many trials may be necessary to reach an individual’s asymptotic discrimination threshold. However, recent research indicates that substantial perceptual learning may occur in the very first trials, as evidenced by the improvements made early in learning by participants [24-27]. Another feature that influences learning tasks is the daily limits of learning. Wright and Sabin [28] observed that training beyond some amount in a single day does not increase the amount of improvement. Therefore, whilst traditional approaches work with long term training, it is important to incorporate early trials into perceptual learning experiments rather than just ignoring them. Although it is accepted that slow perceptual learning is accompanied by enhanced stimulus representation in sensory cortices [29,30], the neural substrates underlying early and rapid improvements are still not fully understood. Recent studies suggest that increased accuracy during the first hour of training may involve increased perceptual sensitivity [31]. Alain et al. [29] showed that the perception of two vowels presented simultaneously could be improved within 1 hour of practice and that improvement coincided with enhancements in an early evoked response (~130ms) localized in the right auditory cortex and a late evoked response (~340ms) localized in the right anterior superior temporal gyrus as well as the inferior prefrontal cortex. Moreover, these learning-related changes were restricted only to participants who attended to the task. The importance of attention in perceptual learning has been reported in many studies as well [21,32-35]. During auditory frequency discrimination, attention seems to play an important role in the process underlying complex auditory tasks, such as comprehension and understanding [36-38]. However, as Jagadeesh [1] discussed in his review it is also possible that plasticity happens in the absence of attention. In this case learning may rely on the inherent salience of the stimulus used to induce plasticity. Attention is drawn implicitly by the stimulus, rather than managed consciously by the individual. Some examples of this type of passive perceptual learning are given in [39] and [40].

To our knowledge, cognitive experiments have investigated changes in brain activity *after* relative to *before* perceptual learning. However, brain activity during perceptual learning has not been explored. We used electrophysiology EEG and functional magnetic resonance imaging fMRI to examine the brain alterations related to fast perceptual learning. In this study we investigate the extent to which enhanced perceptual discrimination results in greater brain activity in modality specific cortex (auditory) to the perceptual event and to what extent frontal regions participate in prediction and top-down modulation of auditory selective attention that gives rise to auditory perceptual learning. For this purpose we designed a paradigm to test auditory frequency discrimination performance during rapid training in which the level of difficulty was based and controlled by an adaptive staircase method. Applying simultaneous EEG and fMRI recording as well as behavioral data, we are able to investigate the underlying sources of activation related to the course of perceptual learning.

## Methods

### Subjects

Simultaneous EEG/fMRI recordings were obtained from 11 subjects (10 males), 22 to 40 years old (mean age 24 years old), with no auditory or visual complaints. Each participant provided informed written consent to participate in the study, which was conducted in accordance with institutional ethical provisions and approved by ATR Human Subject Review Committee in compliance with the Declaration of Helsinki.

### Auditory stimulus

Each auditory stimulus was composed of five tones (400Hz, 600Hz, 700Hz, 800Hz and 1000Hz) with a total duration of 150ms (10ms of rise and fall times) and loudness level of 90 dB SPL. A deviant stimulus differed from the standard in the frequency of the fourth tone. Frequency deviations varied from 1Hz to 40Hz with steps of 1Hz. A sequence of five stimuli was delivered with random ISI ranging from 450 to 500ms. Each sequence had at most one deviant sound on positions 2, 3, 4 or 5. Stimuli were delivered binaurally through a plastic tube attached to foam earplugs using an MRI/EEG compatible system. The tube introduced a constant delay of 64ms in sound presentation to the ears.

### Visual stimulus

Visual stimulus followed the same paradigm. The standard stimulus consisted of a white rectangular horizontal bar positioned in the center of the screen (40cm from the eyes viewed through a mirror). The deviant bars were also positioned in the center but rotated clockwise in steps of 0 to 12 degrees. Stimuli were delivered in sequences of five separated by 450 to 500ms. As in the auditory stimulus presentation, in each sequence of five, there was only one deviant bar and it was never in the first position.

### Behavioral test

Frequency and position discrimination thresholds were measured for each subject in the auditory and visual conditions, separately, in a sound attenuation booth of 40 dBA. The frequency difference between the deviant tones in each trial was changed in a one-up two-down staircase procedure. A staircase is a procedure in which the order of stimulus presentation is determined by responses given by the listener to the trials that were presented previously. In a frequency detection task it provides a method of estimating the signal level that is required for the subject to obtain a particular proportion of correct responses. Therefore, a one-up two-down staircase targets the 71% correct performance level on the psychometric function [41]. In this method the stimulus level is decreased after two positive responses or increased after one negative response in each trial. A positive response requires correctly detecting a deviant in a sequence of five sounds or five bars (in case of visual stimuli). At the end, threshold estimation was done using the arithmetic mean of reversal values [42]. In the visual test, the ability to determine small variations in clockwise rotation of a rectangular bar from horizontal position was tested. The discrimination level obtained in the behavioral test was used as a starting point for the staircase in the MRI experiment.

### 3D scanning

After the behavioral test, a 64 channel electrode cap (BrainCap-MR 64 BrainProducts, Munich, Germany) was placed on the subject. A three dimensional (3D) digitizer (FastScan hand-held laser scanner) was used to acquire subject's head shape and each electrode's position. Surface volumes were later used for source localization procedures.

### Cortical surface model

A polygon cerebral cortex model was constructed using the MRI T_1_ structural image for each subject. The cortical model assumes a current dipole at each vertex at which the fMRI activity elicited by the stimulus exceeded a threshold. The dipole current directions are assumed perpendicular to the cortical surface [43]. Moreover, subjects’ head shapes obtained from the 3D scanner and the structural images were fit using a least squares method. The head was segmented into three compartments: skin, skull and cerebrospinal fluid. Such segmentation was done in Curry software using the boundary element method.

### fMRI experimental design

In the main experiment EEG and fMRI were recorded simultaneously. Stimuli were delivered based on the same staircase procedure used in the behavioral test. A sparse image acquisition technique was applied to prevent contamination of the blood oxygenation level dependent (BOLD) response by the acoustic noise of the scanner and to limit the epochs of contamination of the EEG by the gradient switching during the image acquisition. Functional MRI data were acquired using a Shimadzu Marconi's Magnex Eclipse 1.5T PD250 scanner. Functional data consisted of T_2_*-weighted, gradient echo, echo-planar imaging sequence (TE=48ms and flip angle 90°). During each scan, 165 volumes were acquired over 16.5min. The repetition time (TR) was 6 seconds and the scanning time (TA) was two seconds. Stimulus presentation was made during the “silent” four seconds period. Each volume was composed of 20 axially oriented contiguous slices with 4×4×5mm voxel dimensions with 1mm gap between slices. fMRI data from the first two volumes of each session were discarded to avoid the effects of magnetic saturation. At the end of the experiment a T_1_-weighted structural scan was acquired to align functional data across multiple runs to the subject's reference volume.

**Figure 1:**
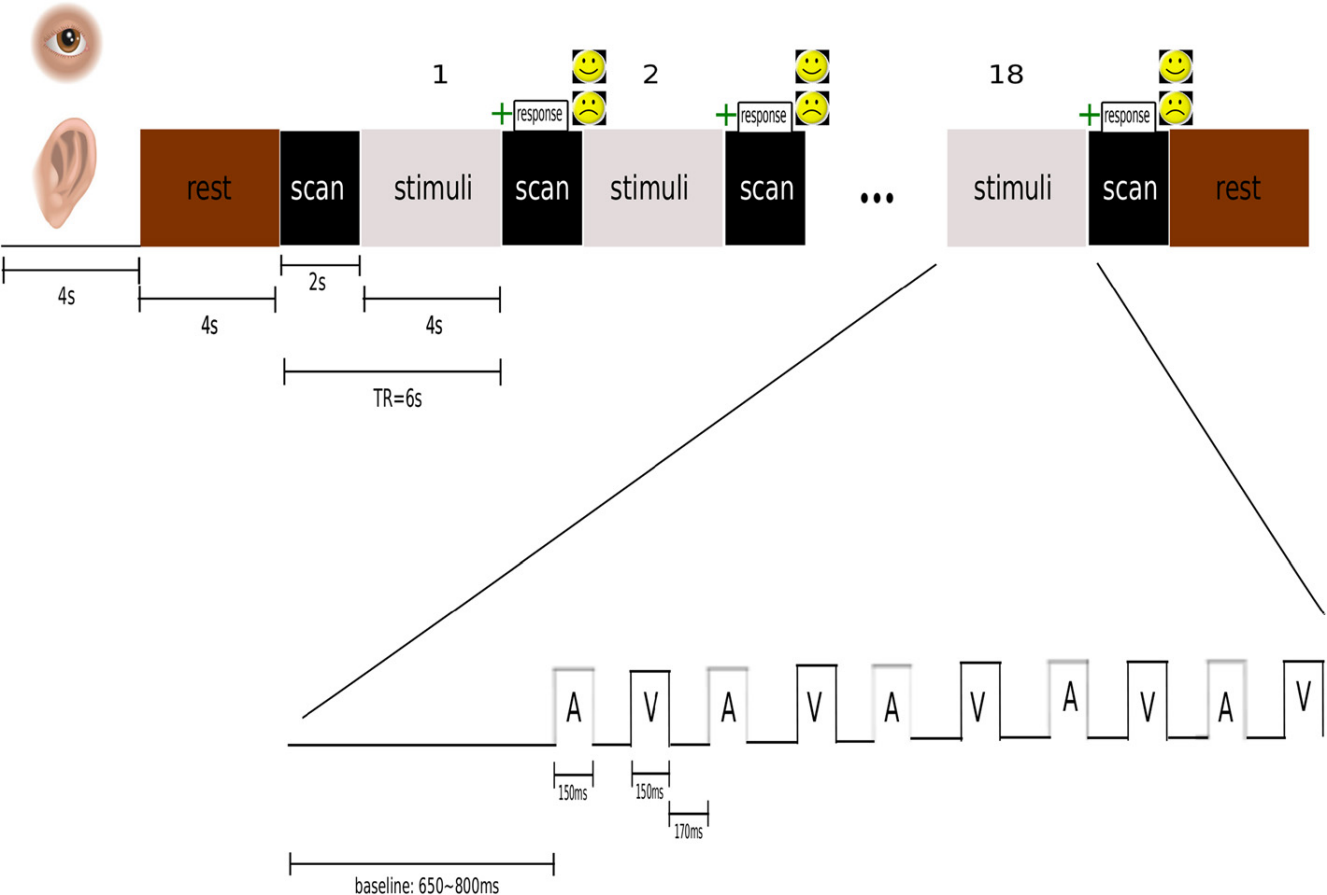
Schematic description of the experimental design.

The experiment was composed of two types of task conditions: auditory and visual. Trials of a single condition were grouped together in blocks of 18 sequences of ten stimuli (five auditory and five visual) lasting 120 seconds in total. Auditory and visual stimuli were interleaved in a sequence separated by a pseudo-random interval ranging from 150 to 175ms. Each block started with a visual instruction in the center of the screen 40cm far from the subject's eyes. Based on what was shown (−Picture of an ear for auditory condition- or -Picture of an eye for visual condition-) the subject had to pay attention to the auditory or visual stimuli. Each instruction lasted four seconds on the screen. Task order was counterbalanced across scanning runs and subjects. Stimuli were delivered during the four seconds of silence when there was no scanning. Before each sequence of stimuli there was a baseline ranging from 650ms to 800ms. After each sequence of 10 stimuli (five visual and five auditory), participants were asked to indicate, by pressing a button (after a green cross appeared on the screen) whether or not a deviant signal was present in the sequence. In this experiment, ‘No’ responses can be either without deviant or with deviant below subject’s perceptual level. A happy face was provided for correct responses, whereas a sad face was presented for incorrect responses. There was a rest condition after each instruction as well as at the end of each block. Figure 1 shows a scheme of the experiment. The recording session consisted of four runs of eight blocks each (four blocks of auditory attention and four blocks of visual attention), resulting in 144 trials acquired per condition per run, with short breaks between them. In this experiment, non-attention to stimulus was attained drawing subject's attention to the other modality (visual or auditory).

### EEG recording

EEG (64-channel) was acquired simultaneously using the Brain Amp MR+fMRI-compatible recorder system in a continuous mode and the BrainCap-MR 64 electrode cap. Potentials recorded at each site were referenced to the center of the head (Cz). Eye movement activity was monitored with an electrode below the left eye. ECG was also recorded simultaneously. The electrode resistance was kept below 5kΩ and the data was sampled at 5kHz per channel.

### Functional image analysis

Analysis was carried out using SPM2 (Wellcome Trust Centre for Neuroimaging, UK). This version was chosen because of the compatibility with VBMEG (source localization procedure). Preprocessing was performed on functional and anatomical images using a common procedure: slice timing, movement correction, normalization and smoothing. Subjects' functional images were coregistered to their own anatomical T_1_ images. Images were spatially normalized to a standard anatomical space defined by a template T_2_ image from the MNI (Montreal Neurological Institute), resampling every 3mm using *sinc* interpolation. Finally, functional images were smoothed with an 8mm FWHM (full-width half maximum) Gaussian kernel. Brain activation during experimental conditions was estimated for each subject using event related fMRI, based on the onset of individual events in the general linear model. Statistical parametric maps were generated for each subject for each experimental condition: auditory response in auditory task (stimulus attended); auditory response in visual task (stimulus unattended) and rest period. Significant voxel activation was determined using *t*-statistics with a threshold of *p*<0.005, uncorrected. To localize brain regions involved in attention demands, activations in the attended and unattended conditions were directly contrasted. In addition, a measure of performance change indicating learning was assessed using the difference between beginning and ending thresholds as a regressor in each session for the auditory-attended condition. It was not possible to investigate the attention related learning effect by doing the analysis over the contrast of the auditory-attended relative to the auditory unattended condition because the auditory unattended condition corresponded to the visual-attended condition in which visual learning was taking place. It becomes somewhat complex to run the modulation of both auditory and visual learning components when learning effects are occurring for both aspects of the contrast of auditory-attended relative to visually-attended (auditory- unattended). Therefore we ran the learning related modulation over the auditory-attended condition only, without subtracting out the visually-attended condition first. To account for performance related variability across subjects, the design matrix was weighted (simple regression analysis) with each subject’s overall gain in a second level analysis.

### EEG data preprocessing

In this study the artifact template subtraction proposed by Allen et al. [44] was used to remove the gradients produced by the switching of magnetic gradients. This approach assumes that the shape of gradient artifacts is constant over time and additive to the physiological signal. Subsequently, independent component analysis (ICA) was conducted over the epoched and baseline removed data (650ms prior to and 3075ms after stimulus onset) in order to extract ballistocardiogram, ocular and movement artifacts [45,46]. The rejection of components was determined by finding a cross-correlation (Pearson’s r>0.3) between each IC and the electrooculogram (EOG) as well as the electrocardiogram (ECG) channels recorded simultaneously with neuronal data. Rejection was also carried out based on abnormal linear trends (using a window width of 932 points, maximum acceptable slope of 0.5 and coefficient of determination R^2^> 0.3). As a final criterion, rejection was carried out by inspecting the components topographic scalp map for characteristics of normal artifact such as eye movement, eye blinks and muscle activity.

The variational hierarchical Bayesian method was used to constrain EEG inverse solutions to regions where fMRI indicates large hemodynamic activation [43,47]. For the estimation, EEG data were divided into 600ms windows with 85% overlap. The prior for each time window was given by the fMRI activity corresponding to the stimulus shown during that time window. The hyperparameters that control the relative amplitude of the prior current variance and the width of the prior distribution were set m_0_=100 and γ_0_=100. The current variance estimation was done using the time sequence of all trials. Each individual’s fMRI activity of all experimental conditions (auditory task attended and unattended) was used as a source localization constraint. For single trial current estimation, the Bayesian inverse filter was applied to three areas of interest determined by using a mask with the learning contrast and extended voxels equal to 50 to clear out areas of no interest.

## Results

### Behavioral data

**Figure 2:**
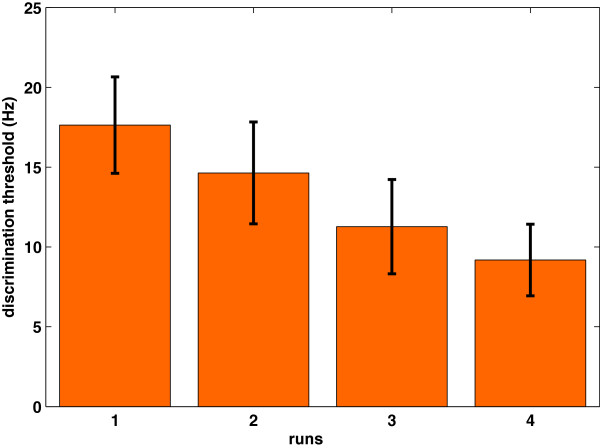
Grand mean and deviant error of 11 subjects for auditory threshold detection at the end of each session.

**Figure 3:**
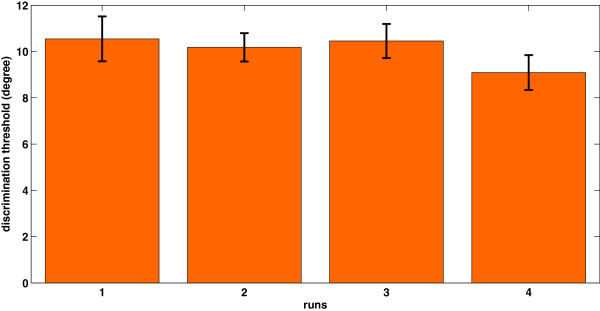
Grand mean and deviant error of 11 subjects for visual threshold detection at the end of each session.

Behavioral data acquired during the experiment shows an exponential, quasi-linear and decreasing tendency in perceptual auditory frequency discrimination thresholds (r=0.99, p=0.0041). Figure 2 shows the grand mean and deviant error of 11 subjects. Although we have used a similar experimental paradigm for the auditory and visual conditions, no behavioral learning effect seems to happen as shown in Figure 3. Given the lack of any behavioral learning effect it is unlikely that the visual stimuli would evoke a visual learning response.

### Functional magnetic resonance imaging

**Figure 4:**
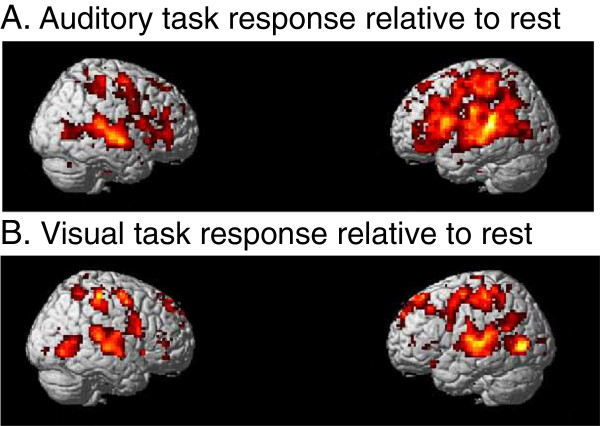
**Result of random-effects fMRI analysis (*p*FDR<0.05). A**. Auditory task condition relative to rest condition. **B**. Visual task condition relative to rest condition.

The brain imaging results of the auditory attended relative to rest contrast show activation in the temporal, frontal and parietal cortices. The auditory unattended (visual attended) relative to rest condition shows activation in parietal, occipital and temporal cortices as summarized in Table 1. Statistical parametric maps for these conditions are given in Figure 4A-B (Auditory: T=2.49, *p*_*FDR*_<0.05, spatial extent threshold=90 voxels; Visual: T=2.66, *p*_*FDR*_<0.05, spatial extent threshold=90 voxels; spatial extent is selected based on uncorrected cluster level p<0.05).

**Table 1:** **Activated areas during auditory and visual stimulation. MNI coordinates of peak activity of clusters (*p*FDR<0.05)**

	**Brain region**	**MNI coordinate**
Auditory vs. rest	Temporal	−48 -3 -27
		48 12 -30
	Frontal	−42 24 39
		33 33 0
	Parietal	−39 -33 42
Visual vs. rest	Occipital	−39 66 6
		−42, -72, 0
		27 -87 -6
		39, -63, 6
	Temporal	−39 -36 15
		36 -33 6
	Parietal	30 -33 39

**Figure 5:**
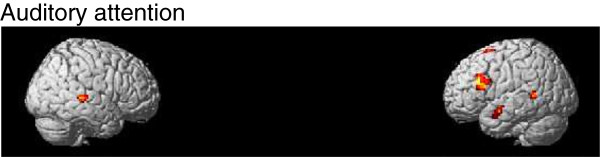
Auditory attentional effect (auditory attented relative to auditory unattended contrast, p<0.005, spatial extent=20 voxels, T=3.17).

**Figure 6:**
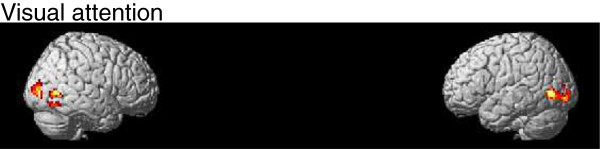
Visual attentional effect (visual attented relative to visual unattended contrast, p<0.005, spatial extent=20 voxels, T=3.11).

With regards to evaluating the attentional load on the task, a direct contrast between auditory attended and auditory unattended (visually attended task) conditions was conducted using the intersection of significant voxels (*p*_FDR_<0.05) of the results given in Figure 4A-B as a mask. Then a small volume correction (SVC) was applied to 6mm radius spherical regions of interest (ROIs) comparing the attention relative to non-attention to the auditory task. The results are shown in Figure 5 and Table 2 with considerable activity (T=3.17) in left inferior frontal gyrus (−45,24,24; *p*_*FDR*_<0.044), left superior temporal gyrus (−57,-51,6; *p*_*FDR*_<0.018 SVC corrected) and right superior temporal gyrus (57,-33,3; *p*_*FDR*_<0.028 SVC corrected). The SVC analyses are based on coordinates given in previous studies of attentional demands (Zhang et al. [48] [−42,13,20]; Kiehl et al. [37] [−62,-34,10]; Zatorre et al. [49] [58,-33,11]). These regions are consistent with sites reported in the literature as reflecting auditory attentional demands. The IFG is considered to be involved with pitch change detection [50,51] and the superior temporal gyrus is a brain region that have been shown to be active in studies investigating auditory short-term functional plasticity [52]. Although our results show stronger hemodynamic responses during the attended condition, Jäncke et al. [52] found a decrease of activation during the course of a 1-week training session. As they reported, one of the reasons for this contradiction might be due to differences with respect to the duration and type of stimulation. While they compare “before” vs. “after” training findings we focus on the responses “during” training. We also analyzed the condition when subject is paying attention to the visual stimuli. Activity in occipital region (Table 3) is higher during attended visual trials (Figure 6) than during attended auditory trials (Figure 5). Previous imaging data have demonstrated that focusing attention on stimuli in one sensory modality increases activity in cortical regions that process stimuli in the attended modality [36,53,54]. Given the lack of any behavioral learning effect it is unlikely that the visual stimuli would evoke a visual learning response. Because of that this paper concerns attention to auditory stimuli only.

**Table 2:** Attentional effect: MNI coordinates of peak activity clusters (T=3.17)

**Brain region**	**MNI coordinate**
Temporal lobe/sub gyral B21	−39, -6, -15
SFG B6	−9, 12, 66
	−9, 3, 63
	−48, 27, 24
MFG B16	−57, 12, 24
IFG B45	57, -33, 3
MTG B22	−57, -51, 6
MTG B22	

**Table 3:** MNI coordinates of peak activity clusters of visual attention (T=3.11)

**Brain region**	**MNI coordinate**
Occipital lobe/ITG	−48, -69, 0
Temporal lobe/ Fusiform gyrus BA37	42, -57, -12
Occipital lobe/ MOG BA19	−30, -87, 15

**Figure 7:**
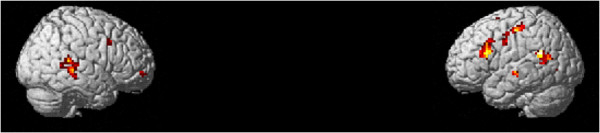
**Learning contrasts weighted by overall gain of each subject (p**_**uncorrected**_**<0.005, spatial extent=20 voxels, T=3.25).**

Since we were interested in assessing learning performance we used the subject’s specific performance gain over each session in the design matrix. The difference between final and initial thresholds was used as regressors in the general linear model for the auditory attended condition. For the second level analysis, intersubject performance differences were accounted for using the overall performance gain as weights in the design matrix. The results are shown in Figure 7 and Table 4 (uncorrected p<0.005). With this procedure we could assess the areas involved in learning as the behavioral data was used as regressors in the data estimation. Small volume correction was performed in the same regions as in Figure 5 with a VOI (volume of interest) of 6mm radius. FMRI activity (T=3.23) were observed in left frontal (−45,15,36; p_FDR_<0.002; SVC corrected), left temporal (−57,-51,24; p_FDR_<0.002; SVC corrected) and right temporal (60,-39,15; p_FDR_<0.001; SVC corrected). The substrates underlying rapid learning-induced changes in the auditory cortex are not yet known but they appear to be concerned with perception and selective attention.

**Table 4:** Learning effect: MNI coordinates of peak activity clusters (T=3.23)

**Brain region**	**MNI coordinate**
Parietal lobe/ postcentral gyrus	−48, -18, 51
	−48, -30, 57
Temporal lobe/ supramarginal gyrus B40	−60, -48, 21
STG B22	−54, -54, 9
MFG B9	−48, 18, 33
	−48, 15, 24
	45, -39, 3
Temporal lobe/ sub-gyral B22	60, -39, 15
STG 22	

### EEG data

**Figure 8:**
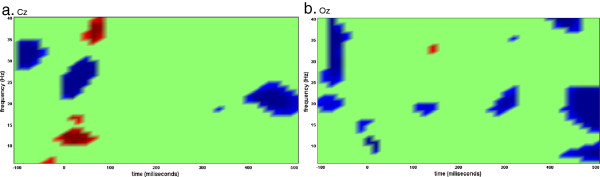
Time frequency representation at Cz for auditory stimulation and Oz for visual stimulation.

Figure 8 shows time frequency plots of scalp site Cz for auditory stimulation and Oz for visual stimulation.

### EEG and fMRI

**Figure 9:**
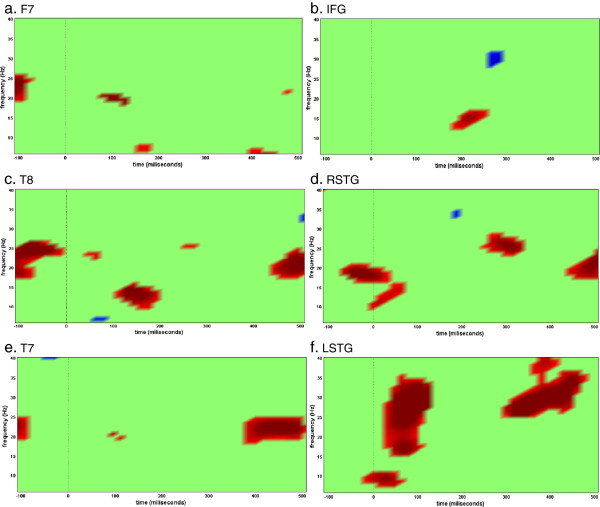
**Statistic tests (p<0.05) carried out on the time-frequency representation of current dipoles in the 3 ROIs analyzed for the auditory versus visual condition. ***t*-test over time-frequency bins of 11 subjects (10 degrees of freedom). Time frequency analysis was done over activity localized on the cortex in **b**) IFG, **d**) RSTG and **f**) LSTG as well as over channel level activity in **a**) F7, **c**) T8 and **e**) T7. In red: bins whose statistics are greater than the null hypothesis of zero mean. In blue: bins whose statistics are smaller than the null hypothesis of zero mean.

Current dipoles were selected within a radius of 6mm from the estimated current peak in each ROI reported in the fMRI analysis (left frontal [IFG: -45,15,36], left temporal [LSTG: -57,-51,24] and right temporal [RSTG: 60,-39,15]). Time frequency analyses were carried out using event-related spectral perturbation ERSP (EEGLAB, [55]) over each of these current dipoles. In this procedure, EEG power within identified frequency bands is displayed relative to power of the baseline period EEG. Blocks of auditory deviant relative to blocks of visual deviant were used to investigate neuronal oscillation at each region of interest. The time-frequency analysis over each current dipole at these areas reveals a different pattern of activation for each subject. Figure 9 shows the statistical results of the attention versus non-attention condition at regions IFG, LSTG and RSTG over activity localized on the cortex as well as at electrodes F7, T7 and T8 for scalp data. The *t*-statistics of all 11 subjects is performed against null hypothesis of zero mean (p<0.05). It can be seen that the responses in LSTG span a wider range compared to the RSTG response, which is more localized in frequency (10 to 20Hz: alpha and beta ranges). The IFG response peaks at around 200ms, later than the temporal cortices as would have been expected. The different responses of neuronal structures in the brain that are frequency band specific have been discussed in the literature in terms of event-related synchronization and desynchronization (ERS/ERD). Quantification of ERS/ERD in time and space has been extensively investigated showing that these responses are functionally related to cognitive processing [56-60]. In this work peak current amplitudes from each region of interest were averaged regardless of phase. This procedure enhanced stimulus-related EEG changes both phase-locked (i.e. event-related potentials) and non-phase-locked (i.e. event-related synchronization and desynchronization) to stimulus onset. Table 5 shows the correlation between EEG power at each frequency band and behavioral threshold at each region of interest (IFG, LSTG and RSTG). Statistical *t*-tests were carried against the hypothesis of null mean at each frequency band. Significant activity were found in IFG at low gamma range (p<0.05 corrected) and marginally non significant in RSTG at beta (p=0.07 corrected) and low gamma (p=0.06 corrected) ranges.

**Table 5:** Mean and standard error of correlation coefficients between Fourier transformed source activity and behavioral threshold values for each subject

	**Alpha**	**Beta**	**Low gamma**	**Gamma**
	(8-13Hz)	(14-28Hz)	(30-35Hz)	(36-40Hz)
IFG	(−0.06, SE=0.08)	(0.17, SE=0.08)	(0.23, SE=0.07)*	(0.05, SE=0.12)
RSTG	(−0.07, SE=0.08)	(0.20, SE=0.08)*	(0.33, SE=0.07)*	(0.15, SE=0.13)
LSTG	(0.07, SE=0.07)	(0.05, SE=0.11)	(0.09, SE=0.08)	(0.04, SE=0.11)

Energy value for each band was computed and correlation between behavioral data was checked. Significant values are marked with (*).

Just for comparison learning analysis was conducted with data at scalp sites F7, T7 and T8 (located above the IFG, LSTG and RSTG respectively). Time-frequency plots of scalp data are shown in Figure 9. Although it is inaccurate to assume that the sensor over an area is mainly reflecting activity just below it we tested the correlation between the energy of each frequency range and behavioral data (Table 6). After correcting for multiple comparisons no significant thresholds are found for the different channels. As can be seen by comparison with the activity source localized to the surface of the cortex there are differences in the mixed activity recorded at the electrodes and the cortical activity in the brain region underneath the electrode.

**Table 6:** Mean and standard error of correlation coefficients between Fourier transformed scalp activity and behavioral threshold values for each subject

	**Alpha**	**Beta**	**Low gamma**	**Gamma**
	(8-13Hz)	(14-28Hz)	(30-35Hz)	(36-40Hz)
F7	(−0.13, SE=0.1)	(0.08, SE=0.11)	(0.12, SE=0.11)	(0.25, SE=0.12)*
T8	(−0.11, SE=0.1)	(0.002, SE=0.09)	(0.14, SE=0.11)	(0.21, SE=0.12)
T7	(−0.07, SE=0.1)	(0.04, SE=0.09)	(0.08, SE=0.11)	(0.22, SE=0.09)*

Energy value for each band was computed and correlation between behavioral data was checked. Significant values are marked with (*).

## Discussion

The results obtained in this study suggest that attention can be involved and contribute to rapid improvements in specific brain activity during short periods of training. Both behavioral and physiological data indicate significant activity for attention specific to auditory task within frontal and temporal areas. We suggest that one component of rapid learning is modulated by selective attention, as evidenced by the engagement with the specific task. Our results fall into the category of early attention theories that support that sensory information being used for processing is modified by attention while non-attended features are discarded [1].

Earlier studies of selective attention [37,61] have shown attention-related enhancements of several auditory evoked electromagnetic signals with early modulation at 20-50ms after stimulus onset. The neural source of this early modulated component has been localized in the posterior part of the superior temporal gyrus. The finding of increased responses to attended auditory stimuli suggests the existence of rapid cortical plasticity. Alain et al. [29] have shown that minutes of classical conditioning are sufficient to induce changes of neural responses and receptive field properties in auditory cortices. This plasticity has also been demonstrated by [62] during an experiment of deafferentation of the adult auditory cortex. Their results show a reorganization of cortical representations occurred within a time period of a few hours. In our work, with approximately 80 minutes of training, an improvement in auditory frequency perception could be observed as the subject’s threshold decreased. These results support the theory that during perceptual learning, a fast improvement, occurring early in training, can be induced by a limited number of trials if specific sensory input is provided.

### Auditory selective attention

The main result of the beta and gamma oscillations found in the study of the correlation between behavioral thresholds and the energy of the current peak values for each trial suggests that plasticity is also manifested as an increase in the power of induced beta and gamma band activity (GBA, >30Hz) in IFG and RSTG (Table 5). The present correlation pattern in IFG and RSTG during attention demands is consistent with findings of gamma band induction during selective attention [63,64]. However, no significant correlation was found for the LSTG. Although GBA enhancements have been reported in multisensory integration [65], selective attention [66] and memory [67] the way these oscillatory synchronizations are involved with cognitive representations is still not fully understood. The reasons for the presence of activity at and before time zero are unclear. One hypothesis of this early response is that it can be a consequence of some form of anticipatory processing [68]. Alternatively it may be a result of the fast stimuli presentation paradigm. At short ISIs the ERP responses to successive stimuli may overlap, distorting the ERP averages. The activity before time zero can, therefore, be a response to previous stimulation. This explanation has been claimed by some researchers to be more plausible than the occurrence of anticipatory phenomena [69].

Moreover, the finding of task related increased activity in frontal and temporal areas is consistent with the hypothesis that the frontal area is involved with prediction and top-down modulation of auditory selective attention that gives rise to auditory perceptual learning. Our current finding of activity in the superior temporal cortices are in accordance with studies that reported enhanced effects of auditory attention in higher association areas when one modality is attended and the other is ignored [36]. Since attentional effects are very dependent on the task, the exact knowledge about the conditions in which the left or right temporal cortices are being activated is still contradictory and deserves further investigation. Rinne et al. [70] and Doeller et al. [71] show evidences of this strong asymmetry in responses with right-hemisphere specialization. In a preattentive auditory deviance processing task, Doeller et al. [71] observed bilateral IFG activation for large compared to medium pitch deviants (50,24,6 (right), -54,26,8(left)). Although most IFG activity during attentional and perceptive tasks are reported in the right hemisphere, left hemisphere activity has also been observed as in [21]. Zhang et al. [48] investigated that the LIFG also serves as a general mechanism for selective attention during a memory task (MNI: -44,15,20; -46,13,21; -42,13,20) as well as Altmann [72] showed LIFG activation when different sound patterns were presented in a sequence of regular sounds (MNI: 47,3,24). Our results show activity enhancement in the superior temporal gyrus as well. Superior temporal gyrus activity has been reported in experiments of attention and perception in the auditory system. Pugh et al. [73] observed a bilateral main effect of attention condition in Brodmann area 22 during a binaural versus dichotic experiment. Right STG (60,-30,11; 58,-33,11) activity was also observed for high and low frequency attended conditions [49]. Looking at the attentional effects (auditory versus visual activity), the modulation role of attention can also been seen in the later responses of IFG peak currents compared to earlier cortical areas such as STG (Figure 9b,d,f). Although the auditory cortices show earlier and stronger responses that can be seen as a bottom-up process, the response in frontal area around 200ms in beta range (14-28Hz) during the auditory attention versus non-attention condition is also evidence of an attentional effect. Moreover, we can see that the difference between VBMEG source activity and data over the sensors F7, T8 and T7 (Figure 9a,c,e) look different because activity under the sensor does not reflect activity of the source underlying the sensor but is a mixture from multiple sources. Whereas, VBMEG localizes activity to specific locations in the brain (IFG, STG and RSTG).

### Gamma and beta range activities

In order to account for learning, we examined the correlation coefficients between time-frequency results in each bin of the attentional responses and the threshold values from the behavioral test for each subject. The results of the group analysis are given in Table 5 (*p*<0.05). In our study we found significant low gamma band induced responses. These results reinforce previous EEG studies showing the involvement of beta and gamma activity in cortical information processing [74]. There is evidence that gamma induced activity is involved in selective attention with enhancement of both the early evoked and later induced gamma-frequency synchronization [75-77]. In our study ERS manifests in IFG and RSTG whereas no significant activity is shown in LSTG. Moreover, the exact role of synchronized gamma activity in attentional processing, as well as the source of these responses, is not yet clear. Correlation was investigated by separating the signal in four frequency ranges: alpha, beta, low gamma and gamma (8-13Hz, 14-28Hz, 30-35Hz, 36-45Hz) and the energy of each range was computed for each trial. The correlation coefficients in Table 5 are sufficient to suggest evidence of correlation, especially in the gamma and beta bands. The significant correlation values in the beta range are consistent with recent results from EEG, MEG and intracortical EEG in humans [78] demonstrating enhanced gamma band oscillatory activity for attended versus unattended stimuli in the auditory cortex [65,79]. Gamma band responses also appear in cortical areas specific to the attended modality during selective attention between visual and auditory modalities [80]. Thus, the early gamma induced response may represent an important processing step related to attention and selection of target stimuli and not only associated to binding processes as previously thought in the visual domain [74,81]. It still needs to be established what mechanism is specific to the beta frequency range. Some authors support the hypothesis that beta activity shifts the system to an attention state (see [82] for visual modality). Haenschel et al. [83] found correlations between gamma and beta activity where evoked gamma oscillations are preceded by beta oscillations in response to novel stimuli. Although our results do not explain the mechanism of these relations beta and gamma activities are significantly correlated to behavioral responses in the attentive modality.

### Control conditions

The STG and IFG have been implicated in several functions beyond that of auditory processing including speech and language processing [84] and social cognition [85]. Our experimental paradigm was carefully designed to account selectively for attention and learning in response to the stimuli presented. To avoid potential confounds caused by anticipation effects the presentation order of the stimuli was randomized. In addition, the time between stimulus presentations was also randomized. To reduce the effects of acoustic noise contamination produced by the fMRI scanning procedure on the cognitive state of the subject we used a sparse presentation procedure in which stimuli were presented in silent periods between scans. To eliminate any biasing effects the same number of deviants and standards were used in the EEG analysis as well as the fMRI analysis. The stimuli themselves did not contain any specific speech, linguistic, or emotion related information that may produce activity in the regions found in our experiment.

In experiments with visual stimulation unconscious involuntary eye movement may be present. These micro-saccades are related to visual fixation and have been shown to have crucial influence on analysis and perception of the visual environment. They can also give rise to EMG eye muscle spikes that can distort the spectrum of the scalp EEG and mimic increases in gamma band power [86]. Some researchers have explored the modulation of synchronous activity by micro-saccades within the primate visual pathway. Yuval-Greenberg et al. [87] have recently noted that spikes in gamma-band activity have a large amount of variability from trial to trial and much of the activity is centered near the eyes. However their results also show a correlation between the amount of gamma band activity and coherence of the image that is shown. In their experiment, during incoherent images micro-saccades were less evident than when the images have some meaning. Melloni et al. [88], however, suggest that saccade related activity is not necessarily trivial and can be related to important cognitive processes that precede, coincide or follow micro-saccades. Recent reports have shown a link between micro-saccades and cognitive processes such as attention, which is not surprising as there is an overlap between the neural systems contributing to control of attention and control of eye movement. There has been a consensus that micro-saccade rates are modulated by both endogenous and exogenous attentional shifts [89]. Additionally, results reporting microsaccades gamma induced activity as being predominantly distributed over the occipital and central scalp [90]. Our results are found in frontal and temporal areas and are not time locked to the onset of the visual stimuli as the control condition was presented randomly.

### The source estimation algorithm

In this work we demonstrated the variational hierarchical Bayesian method proposed by Sato et al. [47] applied to EEG data. The hierarchical variational Bayesian method is a source estimation algorithm that incorporates functional magnetic resonance imaging (fMRI) activity as a hierarchical prior [47,91]. It also incorporates structural MRI data to obtain subject specific information about the position and orientation of the current dipoles. The fMRI information determines the prior distribution of the variance in the cortical current. In the hierarchical Bayesian method, the variance of the cortical current at each source location is considered an unknown parameter and is estimated from the EEG signal by introducing a hierarchical prior on the current variance. Although the first papers with VBMEG demonstrated its applications to MEG data [47,91,92] recent papers have been published since then showing that this technique is appropriate to EEG as well [93]. Aihara et al. [94] applied VBMEG to EEG data by incorporating near-infrared spectroscopy (NIRS) as a hierarchical. VBMEG is, therefore, a multimodal encephalography estimation method.

In this experiment we used VBMEG to get better spatiotemporal resolution that is able to extract localized learning related activity that is mixed at level of sensors. As shown in Table 6 this information can not be obtained from activity recorded at the electrodes as it is inaccurate to assume that the activity at a specific sensor reflects the brain activity just underneath it [95-97].

## Conclusion

The current study explores the advantage of simultaneous fMRI and EEG recording to investigate brain activity during rapid perceptual learning. Behavioral results suggest that listeners can improve quickly at identifying deviant from standard tones. Rapid improvement in task performance is accompanied by plastic changes in the sensory cortex as well as superior areas gated by selective attention. Moreover, the correlation between ERP time-frequency response and results from behavioral test gives support to our hypothesis of learning during short training periods.

## Competing interest

The authors declare that they have no competing interests.

## Authors' contributions

ACSS participated in the design of the study, performed the experiments, processed and analyzed data, collected and drafted the manuscript. HCY contributed to the analyses of the results obtained and revised the manuscript critically. MS provided recommendations for the experimental design as well as the EEG and fMRI simultaneous analysis. DC conceived the study, participated in its design and coordination and edited the manuscript. All authors read and approved the final draft.

## References

[B1] Jagadeesh B Selzer M Clarke SE Cohen LG Duncan PW Gage FH Attentional modulation of cortical plasticity, in Textbook of Neural Repair and Rehabilitation Neural Repair and Plasticity 2006 1 Cambridge: Cambridge University Press 194 205

[B2] Buonomano DV Merzenich MM Cortical plasticity: from synapses to maps Annu Rev Neurosci 1998 21 149 86 10.1146/annurev.neuro.21.1.149 9530495

[B3] Huck JJ Wright BA Late maturation of auditory perceptual learning Dev Sci 2010 13 1 8 10.1111/j.1467-7687.2009.00854.x 21477199 PMC3107547

[B4] Mukai I Kim D Fukunaga M Japee S Marrett S Ungerleider L Activations in visual and attention-related areas predict and correlate with the degree of perceptual learning J Neurosci 2007 27 42 11401 11411 10.1523/JNEUROSCI.3002-07.2007 17942734 PMC6673045

[B5] Kapadia MK Gilbert CD Westheimer G A quantitative measure for short-term cortical plasticity in human vision J Neurosci 1994 14 451 457 8283250 10.1523/JNEUROSCI.14-01-00451.1994 PMC6576842

[B6] Sasaki M Nanez JE Watanabe T Advances in visual perceptual learning and plasticity Nat Rev Neurosci 2010 11 1 53 60 10.1038/nrn2737 19953104 PMC2864603

[B7] Fine I Jacobs RA Perceptual learning for a pattern discrimination task Vision Res 2000 40 23 3209 3230 10.1016/S0042-6989(00)00163-2 11008139

[B8] Schoups AA Vogels R Orban GA Human perceptual learning in identifying the oblique orientation: retinotopy, orientation, specificy and monocularity J Physiol 1995 483 3 797 810 7776259 10.1113/jphysiol.1995.sp020623 PMC1157819

[B9] Westheimer G Crist RE Gorski L Gilbert CD Configuration specificity in bisection acuity Vision Res 2001 41 1133 1138 10.1016/S0042-6989(00)00320-5 11292503

[B10] Ball K Sekuler R A specific and enduring improvement in visual motion discrimination Science 1982 218 697 698 10.1126/science.7134968 7134968

[B11] Yotsumoto Y Watanabe T Sasaki Y Different dynamics of performance and brain activation in the time course of perceptual learning Neuron 2008 57 6 827 833 10.1016/j.neuron.2008.02.034 18367084 PMC2735208

[B12] Banai K Ahissar M Perceptual learning as a tool for boosting working memory among individuals with reading and learning disability Learn. Percept 2009 1 1 115 134 10.1556/LP.1.2009.1.9

[B13] Moore DR Auditory processing disorders: acquisition and treatment J Commun Disord 2007 40 4 295 304 10.1016/j.jcomdis.2007.03.005 17467002

[B14] Musiek FE Shinn J Hare C Plasticity, auditory training and auditory processing disorders Semin Hear 2002 23 263 276 10.1055/s-2002-35862

[B15] Fitzgerald MB Wright B Perceptual learning and generalization resulting from training on an auditory amplitude-modulation detection task J Acoust Soc Am 2011 129 2 898 906 10.1121/1.3531841 21361447 PMC3070992

[B16] Ahissar M Nahum M Nelken I Hochstein S Reverse hierarchies and sensory learning Philos Trans R Soc B 2009 364 285 299 10.1098/rstb.2008.0253 PMC2674477 18986968

[B17] Wright B Zhang Y A review of the generalization of auditory learning Philos Trans R Soc B 2009 364 301 311 10.1098/rstb.2008.0262 PMC2674478 18977731

[B18] Hoare DJ Stacey PC Hall DA The efficacy of auditory perceptual training for tinnitus: a systematic review Ann Behav Med 2010 40 3 313 324 10.1007/s12160-010-9213-5 20668974 PMC2974939

[B19] Flor H Auditory discrimination training for the treatment of tinnitus Appl Psychophysiol Biofeedback 2004 29 2 113 120 15208974 10.1023/b:apbi.0000026637.77671.f4

[B20] King AJ Nelken I Unraveling the principles of auditory cortical processing: can we learn from the visual system? Nat Neurosci 2009 12 6 698 701 10.1038/nn.2308 19471268 PMC3657701

[B21] van Wassenhove V Nagarajan SS Auditory cortical plasticity in learning to discriminate modulation rate J Neurosci 2007 7 2663 2672 17344404 10.1523/JNEUROSCI.4844-06.2007 PMC4096344

[B22] Callan DE Tajima K Callan AM Kubo R Masaki S Akahane-Yamada R Learning-induced neural plasticity associated with improved identification performance after training of a difficult second-language phonetic contrast Neuroimage 2003 19 1 113 124 10.1016/S1053-8119(03)00020-X 12781731

[B23] Demany L Perceptual learning in frequency discrimination J Acoust Soc Am 1985 78 3 1118 1120 10.1121/1.393034 4031256

[B24] Ben-David BM Campeanu S Tremblay KL Alain C Auditory evoked potentials dissociate rapid perceptual learning from task repetition without learning Psychophysiol 2010 48 6 797 807 10.1111/j.1469-8986.2010.01139.x 21054432

[B25] Moore DR Amitay S Hawkey D Auditory perceptual learning Learn Mem 2003 10 83 85 10.1101/lm.59703 12663746 PMC5479143

[B26] Atienza M Cantero JL Dominguez-Marin E The time course of neural changes underlying auditory perceptual learning Learn Mem 2002 9 138 150 10.1101/lm.46502 12075002 PMC182592

[B27] Gilbert CD Early perceptual learning Proc Natl Acad Sci 1994 91 1195 1197 10.1073/pnas.91.4.1195 8108386 PMC43123

[B28] Wright B Sabin A Perceptual learning: how much daily training is enough? Exp Brain Res 2007 180 727 736 10.1007/s00221-007-0898-z 17333009

[B29] Alain C Snyder JS He Y Reinke KS Changes in auditory cortex parallel rapid perceptual learning Cereb Cortex 2007 17 1074 1084 16754653 10.1093/cercor/bhl018

[B30] Bao S Chang EF Woods J Merzenich MM Temporal plasticity in the primary auditory cortex induced by operant perceptual learning Nat Neurosci 2004 7 974 981 10.1038/nn1293 15286790

[B31] Hawkey D Amitay S Moore DR Early and rapid perceptual learning Nat Neurosci 2004 7 1055 1056 10.1038/nn1315 15361880

[B32] Yotsumoto Y Watanabe T Defining a link between perceptual learning and attention PLoS Biol 2008 6 8 1623 1625 10.1371/journal.pbio.0060221 PMC2525694 18752357

[B33] Paffen CLE Verstraten FAJ Vidnyánszky Z Attention-based perceptual learning increases binocular rivalry suppression of irrelevant visual features J Vis 2008 8 4 1 11 10.1167/8.4.1 18484864

[B34] Ahissar M Laiwand R Hochstein S Attentional demands following perceptual skill training Psychol Sci 2001 12 56 62 10.1111/1467-9280.00310 11294229

[B35] Ahissar M Hochstein S Attentional control of early perceptual learning Proc Natl Acad Sci 1993 90 5718 5722 10.1073/pnas.90.12.5718 8516322 PMC46793

[B36] Petkov C Kang X Alho K Bertrand O Yund EW Loods D Attentional modulation of human auditory cortex Nat Neurosci 2004 7 6 658 663 10.1038/nn1256 15156150

[B37] Kiehl KA Laurens KR Duty TL Foster BB Liddle PF An event-related fMRI study of visual and auditory oddball tasks J. Psychophysiol 2001 15 221 240 10.1027//0269-8803.15.4.221 11321614

[B38] Näätanen R The role of attention in auditory information processing as revealed by event-related potentials and other brain measures of cognitive function Behav Brain Sci 1990 13 201 288 10.1017/S0140525X00078407

[B39] Seitz AR Watanabe T Is subliminal learning really passive? Nature 2003 422 36 10.1038/422036a 12621425

[B40] Watanabe T Náñez JE Sasaki Y Perceptual learning without perception Nature 2001 413 844 848 10.1038/35101601 11677607

[B41] Levitt H Transformed up-down methods in psychoacoustics J Acoust Soc Am 1971 49 467 477 10.1121/1.1912375 5541744

[B42] Garcia-Perez M Forced-choice staircases with fixed step sizes: asymptotic and small-sample properties Vision Res 1998 38 1861 1881 10.1016/S0042-6989(97)00340-4 9797963

[B43] Yoshioka T Toyama K Kawato M Yamashita O Nishina S Evaluation of hierarchical bayesian method through retinotopic brain activities reconstruction from fMRI and MEG signals Neuroimage 2008 42 1397 1413 10.1016/j.neuroimage.2008.06.013 18620066

[B44] Allen PJ Josephs O Turner R A method for removing imaging artifact from continuous EEG recorded during functional MRI Neuroimage 2000 12 2 230 239 10.1006/nimg.2000.0599 10913328

[B45] Jung TP Makeig S Westerfield M Analysis and visualization of single-trial event-related potentials Hum Brain Mapp 2002 14 3 166 185 11559961 10.1002/hbm.1050 PMC6871967

[B46] Callan DE Callan AM Kroos C Vatikiotis-Bateson E Multimodal contribution to speech perception revealed by independent component analysis: a single-sweep EEG case study Cogn Brain Res 2001 10 3 349 353 10.1016/S0926-6410(00)00054-9 11167060

[B47] Sato M Yoshioka T Kajiwara S Toyama K Goda N Doya K Kawato M Hierarchical bayesian estimation for MEG inverse problem Neuroimage 2004 23 806 826 10.1016/j.neuroimage.2004.06.037 15528082

[B48] Zhang JX Feng C Fox PT Gao J Tan LH Is left inferior frontal gyrus a general mechanism for selection? Neuroimage 2004 23 596 603 10.1016/j.neuroimage.2004.06.006 15488409

[B49] Zatorre RJ Mondor TA Evans AC Auditory attention to space and frequency activates similar cerebral systems Neuroimage 1999 10 544 554 10.1006/nimg.1999.0491 10547331

[B50] Rinne T Kirjavainen S Salonen O Degerman A Kang X Woods D Alho K Distributed cortical networks for focused auditory attention and distraction Neurosci Lett 2007 416 3 247 251 10.1016/j.neulet.2007.01.077 17368939 PMC2888503

[B51] Alain C Arnott SR Hevenor S Graham S Grady CL “What” and “where” in the human auditory system Proc Natl Acad Sci 2001 98 21 12301 12306 10.1073/pnas.211209098 11572938 PMC59809

[B52] Jäncke L Gaab N Wüstenberg T Scheich H Heinze HJ Short-term functional plasticity in the human auditory cortex: an fMRI study Cogn Brain Res 2001 12 479 485 10.1016/S0926-6410(01)00092-1 11689309

[B53] Mozolic JL Joyner D Hugenschmidt CE Peiffer AM Kraft RA Maldjian JA Laurienti PJ Crossmodal deactivations during modality-specific selective attention BMC Neurol 2008 8 35 10.1186/1471-2377-8-35 PMC2569962 18817554

[B54] Johnson JA Zatorre RJ Attention to simultaneous unrelated auditory and visual events: behavioral and neural correlates Cereb Cortex 2005 15 10 1609 1620 10.1093/cercor/bhi039 15716469

[B55] Delorme A Makeig S EEGLAB: an open source toolbox for analysis of single trial EEG dynamics J Neurosci Methods 2004 134 9 21 10.1016/j.jneumeth.2003.10.009 15102499

[B56] Mu Y Han S Neural oscillations involved in self-referential processing Neuroimage 2010 53 2 757 768 10.1016/j.neuroimage.2010.07.008 20633661

[B57] Basar E Memory and brain dynamics - oscillations integrating attention, perception 2004 Learning and Memory: CRC Press

[B58] Shah AS Bressler SL Knuth KH Ding M Mehta AD Ulbert I Schroeder CE Neural dynamics and the fundamental mechanisms of event-related brain potentials Cereb Cortex 2004 14 5 476 483 10.1093/cercor/bhh009 15054063

[B59] Basar E Schürmann M Toward new theories of brain function and brain dynamics Int J Psychophysiol 2001 39 6 87 89 11163890 10.1016/s0167-8760(00)00134-3

[B60] Pfurtscheller G Lopes-da-Silva FH Event-related EEG/MEG synchronization and desynchronization: basic principles Clin Neurophysiol 1999 110 11 1842 1857 10.1016/S1388-2457(99)00141-8 10576479

[B61] Neelon MF Williams J Garell PC The effects of attentional load on auditory ERPs recorded from human cortex Brain Res 2006 1118 94 105 10.1016/j.brainres.2006.08.006 16956586 PMC2577293

[B62] Pantev C Wollbrink A Roberts LE Engelien A Ütkenhöner B Short-term plasticity of the human auditory cortex Brain Res 1999 842 192 199 10.1016/S0006-8993(99)01835-1 10526109

[B63] Ray S Niebur E Hsiao SS Sinai A Crone NA High-frequency gamma activity (80-150Hz) is increased in human cortex during selective attention Clin Neurophysiol 2008 119 1 116 133 10.1016/j.clinph.2007.09.136 18037343 PMC2444052

[B64] Fan J Byrne J Worden MS Guise KG McCandliss BD Fossella J Posner MI The relations of brain oscillations to attentional networks J Neurosci 2007 27 23 6197 6206 10.1523/JNEUROSCI.1833-07.2007 17553991 PMC6672149

[B65] Kaiser J Hertrich I Ackermann H Lutzenberger W Gamma-band activity over earlysensory areas predicts detection of changes in audiovisual speech stimuli Neuroimage 2006 30 1376 1382 10.1016/j.neuroimage.2005.10.042 16364660

[B66] Kaiser J Lutzenberger W Human gamma-band activity: a window to cognitive processing Neuroreport 2005 16 3 207 211 10.1097/00001756-200502280-00001 15706221

[B67] Lutzenberger W Ripper B Busse L Birbaumer N Kaiser J Dynamics of gamma-band activity during an audiospatial working memory task in humans J Neurosci 2002 22 5630 5638 12097514 10.1523/JNEUROSCI.22-13-05630.2002 PMC6758237

[B68] Linkenkaer-Hansen K Nikulin VV Palva S Ilmoniemi RJ Palva JM Prestimulus oscillations enhance psychophysical performance in humans J Neurosci 2004 24 10186 10190 10.1523/JNEUROSCI.2584-04.2004 15537890 PMC6730198

[B69] Woldorf MG Distortion of ERP averages due to overlap from temporally adjacent ERPs: analysis and correction Psychophysiol 1993 30 98 119 10.1111/j.1469-8986.1993.tb03209.x 8416067

[B70] Rinne T Degerman A Alho K Superior temporal and inferior frontal cortices are activated by infrequent sound duration decrements: an fMRI study Neuroimage 2005 26 1 66 72 10.1016/j.neuroimage.2005.01.017 15862206

[B71] Doeller CF Opitz B Mecklinger A Krick C Reith W Schröger E Prefrontal cortex involvement in preattentive auditory deviance selection Neuroimage 2003 20 1270 1282 10.1016/S1053-8119(03)00389-6 14568496

[B72] Altmann CF Henning M Doring MK Kaiser J Effects of feature-selective attention on auditory pattern and location processing Neuroimage 2008 41 69 79 10.1016/j.neuroimage.2008.02.013 18378168

[B73] Pugh KR Shaywitz BA Shaywitz SE Fullbright RK Skudlarski P Shankweiler DP Katz L Constable RT Fletcher J Lacadie C Marchione K Gore J Auditory selective attention: an fMRI investigation Neuroimage 1996 4 159 173 10.1006/nimg.1996.0067 9345506

[B74] Fell J Fernández G Klaver P Elger CE Fries P Is synchronized neuronal gamma activity relevant for selective attention? Brain Res Rev 2003 42 265 272 10.1016/S0165-0173(03)00178-4 12791444

[B75] Fries P Reynolds JH Rorie AE Desimone R Modulation of oscillatory neuronal synchronization by selective visual attention Science 2001 291 1506 1507 10.1126/science.291.5508.1506 11222864

[B76] Bertrand O Tallon-Baudry C Giard MH Pernier J Auditory induced 40-Hz activity during a frequency discrimination task NeuroImage 1998 7 S370

[B77] Marshall L Mölle M Bartsch P Event-related gamma band activity during passive and active oddball tasks Neuroreport 1996 7 1517 1520 10.1097/00001756-199606170-00016 8856711

[B78] Womelsdorf T Fries P The role of neuronal synchronization in selective attention Curr Opin Neurobiol 2007 17 154 160 10.1016/j.conb.2007.02.002 17306527

[B79] Debener S Herrmann CS Kranczioch C Gembris D Engel AK Top-down attentional processing enhances auditory evoked gamma band activity Neuroreport 2003 14 683 686 10.1097/00001756-200304150-00005 12692463

[B80] Sokolov A Pavlova M Lutzenberger W Birbaumer N Reciprocal modulation of neuromagnetic induced gamma activity by attention in the human visual and auditory cortex Neuroimage 2004 22 521 529 10.1016/j.neuroimage.2004.01.045 15193580

[B81] Jensen O Kaiser J Lachaux J Human gamma-frequency oscillations associated with attention and memory Trends Neurosci 2007 30 7 317 324 10.1016/j.tins.2007.05.001 17499860

[B82] Wróbel A Beta activity: a carrier for visual attention Acta Neurobiol Exp 2000 60 2 247 260 10.55782/ane-2000-1344 10909182

[B83] Haenschel C Baldeweg T Croft RJ Whittington M Gruzelier J Gamma and beta frequency oscillations in response to novel auditory stimuli: A comparison of human electroencephalogram (EEG) data with in vitro models PNAS 2000 97 13 7645 7650 10.1073/pnas.120162397 10852953 PMC16599

[B84] Price C The anatomy of language: a review of 100 fMRI studies published in 2009 Ann N Y Accad Sci 2010 1191 62 88 10.1111/j.1749-6632.2010.05444.x 20392276

[B85] Iacoboni M Dapretto M The mirror neuron system and the consequences of its dysfunction Nat Rev Neurosci 2006 7 12 942 951 10.1038/nrn2024 17115076

[B86] Dimigen O Valsecchi M Sommer W Kliegl R Human microsaccade-related visual brain responses The Journal of Neurosci 2009 29 39 12321 12331 10.1523/JNEUROSCI.0911-09.2009 PMC6666125 19793991

[B87] Yuval-Greenberg S Tomer O Keren AS Nelken I Deouell LY Transient induced gamma-band response in EEG as a manifestation of miniature saccades Neuron 2008 58 3 429 441 10.1016/j.neuron.2008.03.027 18466752

[B88] Melloni L Schwiedrzik CM Rodriguez E Singer W (Micro) Saccades, corollary activity and cortical oscillations Trends Cogn Sci 2009 13 6 239 245 10.1016/j.tics.2009.03.007 19428286

[B89] Martinez-Conde S Macknik SL Troncoso XG Hubel D Microsaccades: a neurophysiological analysis Trends Neurosci 2009 32 9 463 475 10.1016/j.tins.2009.05.006 19716186

[B90] Bosman CA Womelsdorf T Desimore R Fries P A microsaccadic rhythm modulates gamma band synchronization and behavior J Neurosci 2009 29 30 9471 9480 10.1523/JNEUROSCI.1193-09.2009 19641110 PMC6666524

[B91] Toda A Imamizu H Kawato M Sato MA Reconstruction of two-dimensional movement trajectories from selected magnetoencephalography cortical currents by combined sparse Bayesian methods Neuroimage 2011 54 892 905 10.1016/j.neuroimage.2010.09.057 20884361

[B92] Callan DE Callan AM Gamez M Sato M Kawato M Premotor cortex mediates perceptual performance Neuroimage 2010 51 2 844 858 10.1016/j.neuroimage.2010.02.027 20184959

[B93] Yoshimura N DaSalla CS Hanakawa T Sato M Koike Y Reconstruction of flexor and extensor muscle activities from electroencephalography cortical currents Neuroimage 2012 59 1324 1337 10.1016/j.neuroimage.2011.08.029 21945691

[B94] Aihara T Takeda Y Takeda K Yasuda W Sato T Otaka Y Hanakawa T Honda M Liu M Kawato M Sato M Osu R Cortical current source estimation from electroencephalography in combination with near-infrared spectroscopy as a hierarchical prior Neuroimage 2012 59 4006 4021 10.1016/j.neuroimage.2011.09.087 22036684

[B95] Nunez P Neocortical Dynamics and Human EEG Rhythms 1995 New York: Oxford University Press

[B96] Michel CM Murray MM Lantz G Gonzalez S Spinelli L Michel CM Murray MM Lantz G Gonzalez S Spinelli L Grave De Peralta R EEG source imaging Clinical Neurophysiol 2004 115 2195 2222 10.1016/j.clinph.2004.06.001 15351361

[B97] Nunez P Srinivasan R Electric Fields of the Brain: The Neurophysics of EEG 2006 New York: Oxford University Press

